# Ligation of Subclavian and of Common Carotid Artery for Aneurisms

**Published:** 1889-08

**Authors:** W. J. Pettus

**Affiliations:** Assistant Surgeon U. S. Marine Hospital Service, Lecturer on Diseases of the Skin, Genito Urinary Organs, and Venereal Diseases, Texas Medical College and Hospital, Galveston, Texas


BY W. J. PETTUS, M. D.,

Assistant Surgeon U. S. Marine Hospital Service, Lecturer on Diseases of the Skin, Genito Urinary Organs, and Venereal Diseases, Texas Medical College and Hospital, Galveston, Texas.

CASE I.—ANEURISM OF INNOMINATE AND RIGHT CAROTID ARTERIES, LIGATION, OF COMMON CAROTID AND SUB-CLAVIAN; IMPROVEMENT.

MRS. M. C., age 40 years, Irish, never seriously ill before, but addicted to drinking. Four years ago, she noticed a sensation of accelerated beating of the heart, and a feeling of oppression extending over the left side, palpitation sometimes interfered with sleep; voice became husky, respiration on exertion became rapid and difficult. January, 1889, a pulsating tumor, visible from a distance, filling the supra-sternal groove and reaching up in front of right sterno-mastoid muscle. By touch a pulsating sac was felt, corresponding to the innominate and lower part of the carotid, and probably extending down into, and involving the arch of the aorta. This sac possesses all the characteristic signs of an aneurism; loud bruit heard, with a distinct thrill; apex beat of heart, between 6th and 7th ribs, otherwise normal condition of heart; diagnosis, aneurism, involving the innominate and common carotid, and probably the arch of the aorta, due to atheromatous degeneration. Operation by Drs. Pettus and Hadra. Ligation of right common carotid, above omo-hyoid muscle, and of right sub-clavian in the third part. Pulsation at wrist at once disappeared. Patient rallied well from chloroform, and had not the slightest symptoms of cerebral disturbance. Some oversight in asepsis must have been the cause of the tedious healing of the wound at the site of the ligation of sub-clavian artery. Catgut ligature used on both vessels. The pulsation of the supra-sternal part of the aneurism was but little diminished immediately after the operation, and after a few days seemed to be as strong, or even a little stronger than before, but patient expressed herself as feeling much better; her former disagreeable and painful sensation being much improved. Patient re-examined about July 1st, 1889; pulsation and size of sac very much diminished in the supra-sternal groove; carotid portion of aneurism also much smaller. Patient is very much improved, rests easily, suffers scarcely at all from palpitation and shortness of breath, and is rid of the severe, gnawing pain felt between the shoulders. Before the operation, she was unable to pursue her occupation, that of a cook, but now is working with much ease.

### CASE 2.—ANEURISM OF RIGHT COMMON CAROTID ARTERY, LIGATION OF ARTERY, RECOVERY.

Chas. Richardson, age 51, American, sailor, of intemperate habits, came under observation on March 29th, 1889, complaining of toothache, or rather neuralgia of right side of the face. He stated that the toothache had caused the swelling, visible on the right side of his neck, both having come on at the same time, eight days before. Examination disclosed a rounded swelling, extending from about 1½ inches below the ear to the same distance above the clavicle. Palpation showed a marked pulsation and thrill over the sac, and upon consultation a loud and distinct bruit could be heard. He complained of great dryness of the throat, with a harsh, dry cough, could not speak above a whisper, and had great difficulty in swallowing. He was admitted to the U. S. Marine wards on March 30th, and on April 1st, the carotid was ligated by Dr. Pettus with a boiled silk ligature, below the omo-hyoid. The sac at once became much smaller, and pulsation disappeared entirely. On April 6th, the dressing was removed, showing union by first intention, except where drainage tube was introduced. On May 6th, faint, but distinct pulsation could be felt in the sac, but on the 13th of the same month, he was discharged recovered. Examination on July 5th, showed a complete recovery, there being no bruit or thrill to be heard or felt, and the position of the former aneurism, could only be told by a small hard lump, easily felt.

GALVESTON, TEXAS, July 27, 1889.

